# EXAMINING SOCIOCULTURAL RESILIENCE FACTORS IN WIDOWHOOD AND COGNITIVE FUNCTION AMONG OLDER CHINESE IMMIGRANTS

**DOI:** 10.1093/geroni/igad104.0828

**Published:** 2023-12-21

**Authors:** Yura Lee, Yanping Jiang

**Affiliations:** University of Wisconsin-Milwaukee, Milwaukee, Wisconsin, United States; Rutgers University, New Brunswick, New Jersey, United States

## Abstract

Loss of a spouse is one of the most stressful life events in later life and often entails significant physical and mental health outcomes for widowed individuals. Although there is growing evidence on widowhood and cognitive function, existing studies have shown mixed results. Little is known about resilience factors that may attenuate the adverse effect of widowhood on cognition among older Asian immigrants. This study explored potential moderators (i.e., social support, acculturation, leisure activities), which may contribute to resilience in the association between widowhood and cognitive function among older Chinese immigrants. The study sample included 2,515 adults aged 60 or older who completed two waves (2011–2013 and 2013–2015) of the Population Study of Chinese Elderly in Chicago. Cognitive function was measured by global cognitive function and episodic memory. Widowhood group was categorized into three groups based on the marital status at two waves: 1) married, 2) recently widowed and 3) previously widowed. Linear regression analyses were conducted with interaction terms. Our results show that social support moderated the relationship between widowhood and global cognitive function, and acculturation moderated the relationship between widowhood and episodic memory. Specifically, the findings indicate that adverse effect of widowhood on cognitive function is more pronounced at lower levels of social support and acculturation. This implies buffering roles of social support and acculturation in cognitive health among older Chinese immigrants who experience widowhood. Providing supportive programs and interventions to increase social support and acculturation is suggested to promote cognitive function in this population.

